# Gene Expression Profile at the Motor Endplate of the Neuromuscular Junction of Fast-Twitch Muscle

**DOI:** 10.3389/fnmol.2020.00154

**Published:** 2020-09-08

**Authors:** Kun Huang, Jin Li, Mikako Ito, Jun-Ichi Takeda, Bisei Ohkawara, Tomoo Ogi, Akio Masuda, Kinji Ohno

**Affiliations:** ^1^Division of Neurogenetics, Center for Neurological Diseases and Cancer, Nagoya University Graduate School of Medicine, Nagoya, Japan; ^2^Department of Genetics, Research Institute of Environmental Medicine (RIeM), Nagoya University, Nagoya, Japan

**Keywords:** neuromuscular junction, motor endplate, collagen Q, ribotag mouse, gene expression profile

## Abstract

The neuromuscular junction (NMJ) is a prototypic chemical synapse between the spinal motor neuron and the motor endplate. Gene expression profiles of the motor endplate are not fully elucidated. Collagen Q (ColQ) is a collagenic tail subunit of asymmetric forms of acetylcholinesterase and is driven by two distinct promoters. p*ColQ1* is active throughout the slow-twitch muscle, whereas p*ColQ1a* is active at the motor endplate of fast-twitch muscle. We made a transgenic mouse line that expresses nuclear localization signal (NLS)-attached Cre recombinase under the control of p*ColQ1a* (p*ColQ1a*-Cre mouse). RiboTag mouse expresses an HA-tagged ribosomal subunit, RPL22, in cells expressing Cre recombinase. We generated p*ColQ1a*-Cre:RiboTag mouse, and confirmed that HA-tagged RPL22 was enriched at the NMJ of tibialis anterior (TA) muscle. Next, we confirmed that *Chrne* and *Musk* that are specifically expressed at the NMJ were indeed enriched in HA-immunoprecipitated (IP) RNA, whereas *Sox10* and *S100b*, markers for Schwann cells, and *Icam1*, a marker for vascular endothelial cells, and *Pax3*, a marker for muscle satellite cells, were scarcely detected. Gene set enrichment analysis (GSEA) of RNA-seq data showed that “phosphatidylinositol signaling system” and “extracellular matrix receptor interaction” were enriched at the motor endplate. Subsequent analysis revealed that genes encoding diacylglycerol kinases, phosphatidylinositol kinases, phospholipases, integrins, and laminins were enriched at the motor endplate. We first characterized the gene expression profile under translation at the motor endplate of TA muscle using the RiboTag technique. We expect that our gene expression profiling will help elucidate molecular mechanisms of the development, maintenance, and pathology of the NMJ.

## Introduction

**Figure 1:**
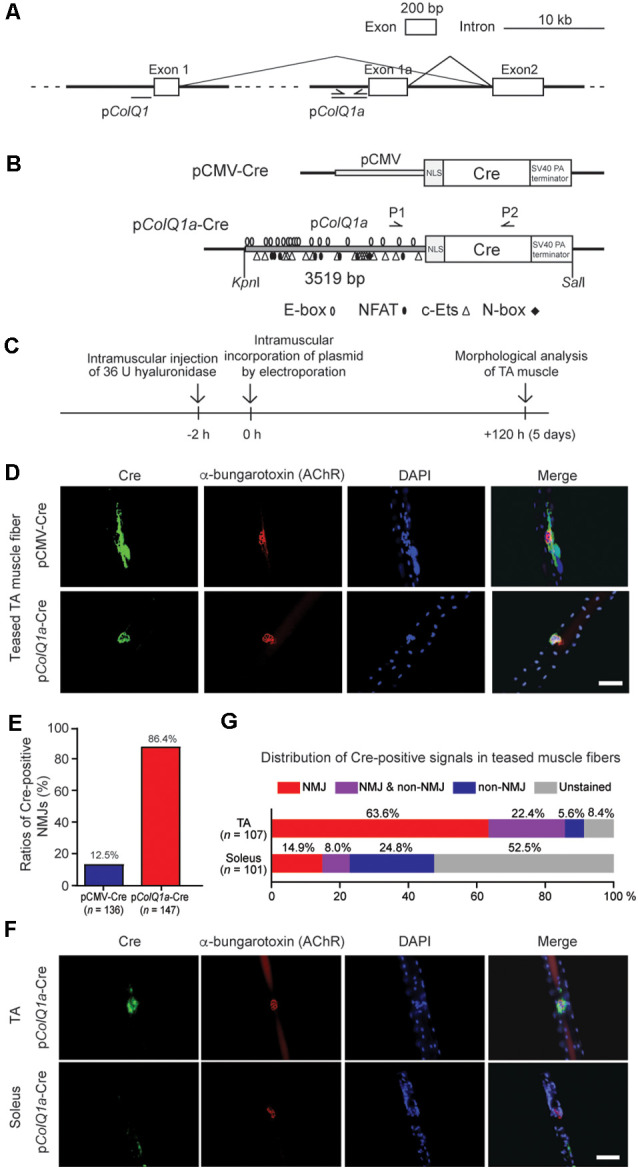
Mouse p*ColQ1a* promoter expresses Cre at the neuromuscular junction (NMJ) of the tibialis anterior (TA) muscle. **(A)** Mouse *Colq* is driven by two distinct promoters. p*ColQ1* drives transcription from exon 1, whereas p*ColQ1a* drives transcription from exon 1a. PCR primers (Supplementary Table S1) to amplify a 3519-bp fragment of p*ColQ1a* are indicated by arrows. **(B)** Schematic of pCMV-Cre and p*ColQ1a*-Cre. pCMV or p*ColQ1a* was fused to the nuclear localization signal (NLS) sequence and the Cre recombinase gene. P1 and P2 arrows indicate the positions of genotyping primers. Putative binding motifs of transcription factors (E-box, NFAT, c-Ets, and N-box) are indicated (Lee et al., 2004). **(C)** The protocol of muscle electroporation. **(D)** Representative immunostaining of Cre (green) and Alexa594-conjugated α-bungarotoxin-staining for AChR (red) of teased TA muscle fibers at 5 days after electroporation of the indicated plasmids. The nuclei were stained with DAPI. Scale bar = 30 μm. **(E)** Ratios of Cre-positive NMJs in teased TA muscle fibers electroporated with pCMV-Cre and p*ColQ1a*-Cre in 4 pCMV-Cre mice (*n* = 136 fibers, respectively) and 5 p*ColQ1a*-Cre mice (*n* = 147 fibers). *P* < 0.001 by Fisher’s exact test (see Supplementary Table S3 for individual counts). **(F)** Representative images showing NMJ-specific expression of Cre in teased TA muscle fibers but not in teased soleus muscle fibers of the p*ColQ1a*-Cre mouse. Teased muscle fibers were stained with anti-Cre antibody (green) and Alexa594-conjugated α-bungarotoxin 594 (red). Nuclei were stained with DAPI. Scale bar = 30 μm. **(G)** Distribution of Cre-positive signals along teased muscle fibers of TA and soleus muscles of the p*ColQ1a*-Cre mouse. The total number of teased muscle fibers is shown in parentheses. *P* < 0.001 by Fisher’s exact test (see Supplementary Table S4 for individual counts).

Neuromuscular junction (NMJ) is a prototypic synapse between the spinal motor neuron and the motor endplate (Hirsch, 2007; Ohno et al., 2017). At the NMJ, acetylcholinesterase (AChE) rapidly hydrolyzes acetylcholine (ACh) released from the nerve terminal (Ohno et al., 1998; Gaspersic et al., 1999). At the NMJ, a globular form of AChE makes a homomeric tetramer. One, two, and three tetrameric AChE are bound to triple-helical collagen Q (ColQ) to make asymmetric A_4_, A_8_, and A_12_ forms, and are anchored to the synaptic basal lamina (Nazim et al., 2017). Transcription of *Colq* is driven by two distinct promoters of p*ColQ1* and p*ColQ1a* (Figure 1A; Krejci et al., 1999; Lee et al., 2004; Ting et al., 2005). p*ColQ1* is active throughout the slow-twitch muscle. p*ColQ1* starts transcription from *Colq* exon 1 to generate A_4_ and A_8_ forms. In contrast, p*ColQ1a* is active at the NMJ, but not at the extrajunctional regions, of the fast-twitch muscle. p*ColQ1a* starts transcription from *Colq* exon 1a to predominantly generate A_12_ form. Human p*ColQ1a* carries consensus sequences for transcriptional factors E-protein (E-box, CANNTG), NFAT (GGAAA), c-Ets transcription factor [c-Ets, (C/A)GGA(A/T)], Elk-1, N-box (CCGGAA), and MEF2 (CTAAAAATAA), which play essential roles in muscle-specific and NMJ-specific transcriptional activities (Lee et al., 2004).

Massive parallel sequencing of RNA (RNA-seq) enabled the identification of gene expression profiles in specific tissues and cells at an unprecedented level of accuracy. Also, tissue-specific and cell-type-specific transcriptomic profiling is made available by fluorescence-activated cell sorting (FACS), laser capture microdissection, and single-cell RNA-seq (scRNA-seq; Kim et al., 2015). However, gene expression profiles can be artificially changed in the course of FACS and scRNA-seq procedures. In laser capture microdissection, target cells are frequently contaminated by surrounding cells, because target cells should be free from laser burns. RiboTag mouse technique was developed to circumvent these disadvantages (Sanz et al., 2009). RiboTag mouse carries an RPL22 subunit of the ribosome with a hemagglutinin tag at the C-terminal end (RPL22-HA), and its expression is induced by Cre recombinase (Sanz et al., 2009). Cell-type-specific expression of Cre recombinase thus enables isolation of total mRNA under translation in a cell-type-specific manner.

In our study, we first generated a transgenic p*ColQ1a*-Cre mouse that specifically expresses Cre recombinase in the subsynaptic nuclei of fast-twitch muscle. As far as we know, no transgenic mouse has been generated to express Cre recombinase at the motor endplate. We confirmed that Cre recombinase is highly expressed at the NMJ of fast-twitch muscle, but not of slow-twitch muscle, in the p*ColQ1a*-Cre mouse. To characterize gene expression profiles at the motor endplate of fast-twitch muscle, we made p*ColQ1a*-Cre:RiboTag mouse that expresses RPL22-HA only at the motor endplate. Quantitative RT-PCR (qRT-PCR) showed that mRNA immunoprecipitated with the HA tag from tibialis anterior (TA) muscle of p*ColQ1a*-Cre:RiboTag mouse was enriched for motor endplate-specific genes, and mostly depleted for genes specific for Schwann cells and vascular endothelial cells. RNA-seq analysis of HA-immunoprecipitated mRNA revealed enrichment of genes encoding diacylglycerol kinases, phosphatidylinositol kinases, phospholipases, integrins, and laminins.

## Materials and Methods

### Construction of Plasmids

A Cre cDNA originating from Type I topoisomerase of bacteriophage P1 fragment was amplified from a pAAV-Cre plasmid (AAV-401, Cell Biolabs). The nuclear localization signal (NLS) of the SV40 large T antigen sequence (5’-GAGCACCATGCCAAAAAAGAAGAGAAAGGTATCCAATTTAC-3’) was synthesized by Sigma–Aldrich. A 3519-bp mouse p*ColQ1a* promoter (positions 31,557,371–31,553,853 on chromosome 14 according to GRCm38/mm10) was amplified by PCR using genomic DNA of C57BL/6 mouse. We ligated p*ColQ1a*, NLS, and Cre cDNA to generate p*ColQ1a*-Cre (Figure 1B). As a control, we generated pCMV-Cre. Primers for PCR are shown in Supplementary Table S1. Plasmids were purified with the EndoFree Megaprep DNA Purification System (Qiagen). The absence of artifacts in the plasmid inserts was confirmed by Sanger sequencing.

### Immunofluorescent Staining

TA and soleus muscles were harvested and fixed in 4% paraformaldehyde (PFA) for 1 h and were teased into muscle fibers in phosphate-buffered saline (PBS). The teased fibers were incubated in 100 mM glycine in PBS for 15 min at room temperature (RT). The fibers were washed in PBS three times for 15 min each and were incubated in a blocking buffer (2% bovine serum albumin, 5% goat serum, and 0.5% Triton X-100 in PBS) for 1 h at RT. After blocking, the fibers were incubated with mouse monoclonal anti-Cre recombinase antibody (1:100, MAB3120, Millipore) or anti-HA antibody (1:200, Covance) in a blocking buffer overnight at 4°C. After washed in 0.1% Triton X-100 in PBS several times, the fibers were incubated with FITC-conjugated goat anti-mouse IgG (1:100, FI2000, Vector Laboratories). AChR was stained with Alexa 594-conjugated α-bungarotoxin (1:500, B13423, Invitrogen) for 2 h at RT. Finally, the fibers were covered by a coverslip with Vectashield with 4’,6-diamidino-2-phenylindole (DAPI; Vector Laboratories). Fluorescence signals were quantified by the IX71 microscope (Olympus) equipped with the MetaMorph imaging software (Molecular Devices).

Frozen sections of TA muscle at 10 μm were incubated with a primary antibody (anti-HA antibody at 1:200, Covance) overnight at 4°C. The sections were added with FITC-conjugated goat anti-mouse IgG (1:100, FI2000, Vector Laboratories) and Alexa 594-conjugated α-bungarotoxin (1:500, B13423, Invitrogen) followed by incubation for 1 h at RT. Finally, the sections were covered by a coverslip with Vectashield with DAPI, and fluorescence signals were quantified as stated above.

### Electroporation of Plasmids Into Mouse TA and Soleus Muscle

All mouse experiments were approved by the Animal Care and Use Committee of the Nagoya University Graduate School of Medicine and were performed following the relevant guidelines. Under deep anesthesia of a 6-week-old C57BL/6 mouse, 36 U hyaluronidase (H4272, SIGMA) was injected into the TA muscle 2 h before injection of pCMV-Cre or p*ColQ1a*-Cre. Then, 50 μl of each plasmid (1,000 ng/μl) was injected into the TA or soleus muscle and applied electrical pulses at 50 volts. The electric pulses were delivered in parallel to the muscle fibers with forceps-shaped electrodes (CUY650P3, Nepa Gene) connected to an electric pulse generator (NEPA21, Nepa Gene). The electrical pulses were comprised of three pulses of a rectangular wave for 25 ms at a rate of 3 Hz, followed by three pulses of the opposite polarity with the same protocol. TA or soleus muscle was harvested on day 5 after electroporation.

### Generation of p*ColQ1a*-Cre Mouse

A fragment of p*ColQ1a*-Cre (Figure 1B) was microinjected into fertilized eggs of C57BL/6 mouse. The microinjection and subsequent transfer to foster mothers were technically supported by the Division of Experimental Animals, Nagoya University Graduate School of Medicine.

### Generation of p*ColQ1a*-Cre:RiboTag Mouse

RiboTag mice were kindly provided by Dr. Taiji Matsusaka at Tokai University under the permission of Dr. Paul S. Amieux at Western Washington University (Sanz et al., 2009). RiboTag mouse was mated to p*ColQ1a*-Cre mouse to obtain p*ColQ1a*-Cre:RiboTag mouse.

### Immunoprecipitation Assays

Immunoprecipitation (IP) of pooled TA muscles of 6-week-old p*ColQ1a*-Cre:RiboTag mice was performed as described previously with minor modifications (Sanz et al., 2009). Briefly, TA muscle was mashed by FastPrep 24 Instrument (MBP) in an IP buffer (50 mM Tris-HCl, pH 7.5, 100 mM KCl, 12 mM MgCl_2_, 1% Nonidet P-40, 1 mM DTT, 400 units/ml Toyobo RNase Inhibitor, 1 mg/ml heparin, 100 μg/ml cycloheximide, and the cOmplete protease inhibitor (one tablet in 25 ml, Roche). Then, anti-HA antibody (1:300, Covance) was added to 1,200 μl of cleared homogenates and incubated for 6 h at 4°C. Then protein G magnetic beads (Dynabeads, Invitrogen) in an IP buffer were added and incubated overnight at 4°C. After incubation, beads were washed four times for 5 min in a high-salt buffer (50 mM Tris-HCl, pH 7.5, 300 mM KCl, 12 mM MgCl_2_, 1% Nonidet P-40, 1 mM DTT, and 100 μg/ml cycloheximide) in the presence of 400 units/ml RNase Inhibitor (Toyobo) and the cOmplete™ protease inhibitor (one tablet in 25 ml, Roche). RNA was eluted in an RLT buffer with β-mercaptoethanol (Qiagen) and was purified by RNeasy Micro Kit (Qiagen) with DNase treatment. Total RNA was quantified and its quality was evaluated by RNA integrity number (RIN; Schroeder et al., 2006) representing the quality and integrity of RNA using an Agilent 2100 Bioanalyzer with the RNA 6000 Pico kit (Agilent Technologies).

### Gene Expression Analysis

After confirming that the RIN was higher than 7.0, total RNA was reverse-transcribed using random hexamer primers (Thermo Fisher Scientific) and ReverTra Ace (Toyobo). Quantitative RT-PCR (qRT-PCR) was performed with the LightCycler 480 (Roche Applied Science) using the TB Green Premix ExTaq II (Takara Bio). Gene expression levels were normalized to that of glyceraldehyde-3-phosphate dehydrogenase (*Gapdh*), and also to the ratio of input RNA. For both IP RNA and input RNA, TA muscles of 10, 11, 12, and 16 p*ColQ1a*-Cre:Ribotag mice were pooled into four independent samples to be analyzed in quadruplicate. qRT-PCR primers are indicated in Supplementary Table S2.

### High-Throughput RNA Sequencing (RNA-Seq)

In the four pooled pairs of IP RNA and input RNA samples stated above, a pair of samples arising from 11 p*ColQ1a*-Cre:Ribotag mice had the highest RIN in both IP RNA and input RNA, and was subjected to RNA-seq. Quality of the RNA samples was checked using both an Agilent TapeStation and an Agilent 2100 Bioanalyzer with the RNA 6000 Nano Kit, and the following thresholds were applied: quantity (>100 ng), concentration (>1 ng/μl), no contamination of DNA, no degradation of RNA confirmed by rRNA peaks (18S, 28S), and RIN (>7.0). RNA-seq of Input RNA and IP RNA was performed at Macrogen and BGI, respectively. At both companies, a sequencing library was prepared using the TruSeq Stranded mRNA kit (Illumina), and the library was read on Illumina NovaSeq 6000 (150 bp paired-end reads). As the amount of IP RNA remained low even after pooling TA muscles, IP RNA was linearly amplified by the Smart-seq2 protocols (Picelli et al., 2014). Raw reads were mapped to the mouse reference genome (NCBI37/mm9) using STAR version 2.5.3a (Dobin et al., 2013) with standard options for the long RNA-seq pipeline by ENCODE (Consortium, 2012). Transcripts per million (TPM) of each gene was calculated by RSEM version 1.3.1 with default parameters (Li and Dewey, 2011).

### Gene Set Enrichment Analysis (GSEA)

We performed pathway analysis with Gene Set Enrichment Analysis (GSEA^1^; Vickers et al., 2008) using the following parameters: the number of permutations was 1,000; the phenotype labels were “IP vs. input;” the collapse/remap to gene symbols was “collapse;” the permutation type was configured to the “gene set” to avoid the potential problem of small sample size; and the chip platform was “Mouse Gene Symbol Remapping to Human Orthologs MSigDB.v7.1.chip.” The KEGG subset of canonical pathways (186 gene sets) was used as a reference gene set. Nominal *p*-value <0.05 and false-discovery rate (FDR) *q-value* <0.25 were used as the cut-off criteria (Reimand et al., 2019).

## Results

### Mouse p*ColQ1a* Promoter Expresses Cre at the NMJ of the TA Muscle

Transcriptions of the mouse *ColQ* gene start from exons 1 and 1a, which are driven by p*ColQ1* and p*ColQ1a* promoters, respectively (Figure 1A). A 3519-bp DNA fragment of mouse p*ColQ1a* was amplified by PCR and was added 5′ to the nuclear localization signal (NLS) and Cre cDNA to generate a p*ColQ1a*-Cre plasmid (Figure 1B). As the skeletal muscle fibers are comprised of multi-nucleated cells, NLS-mediated confinement of Cre in the nuclei was essential.

To confirm that p*ColQ1a*-Cre expresses Cre specifically in the subsynaptic nuclei of TA muscle, we introduced p*ColQ1a*-Cre in the mouse hindlimb by electroporation (Figure 1C; Murakami et al., 2003; Young and Dean, 2015). pCMV-Cre was used as a control. The TA and soleus muscles were teased into single fibers to visualize the NMJs. pCMV-Cre expressed Cre throughout TA muscle, and only 12.5% of Cre-positive areas were overlapped with AChR-positive areas. In contrast, p*ColQ1a*-Cre expressed Cre at the NMJ of TA muscle, and 86.4% of Cre-positive areas were overlapped with AChR-positive areas (Figures 1D,E and Supplementary Table S3). These results indicate that the p*ColQ1a* promoter drove the subsynaptic expression of Cre in the TA muscle.

Next, we generated a transgenic mouse line carrying p*ColQ1a*-Cre. The expression of Cre was again examined in teased muscle fibers of the TA and soleus muscles, which are mostly comprised of fast-twitch and slow-twitch muscle fibers, respectively (Figure 1F). In TA muscle, 63.6% of teased muscle fibers expressed Cre at the NMJ. In contrast, in soleus muscle, the ratio was only 14.9%. Notably, 52.5% of the soleus muscle fibers did not express Cre either at the NMJ or at the extrajunctional regions (Figure 1G and Supplementary Table S4). These results indicate that the 3519-bp fragment of the mouse p*ColQ1a* promoter region restricted the expression of Cre at the NMJs of fast-twitch muscle but not of slow-twitch muscle.

### p*ColQ1a*-Cre:Ribotag Mouse Expresses HA-Tagged Ribosomal Protein RPL22 at the NMJ of TA Muscle

**Figure 2:**
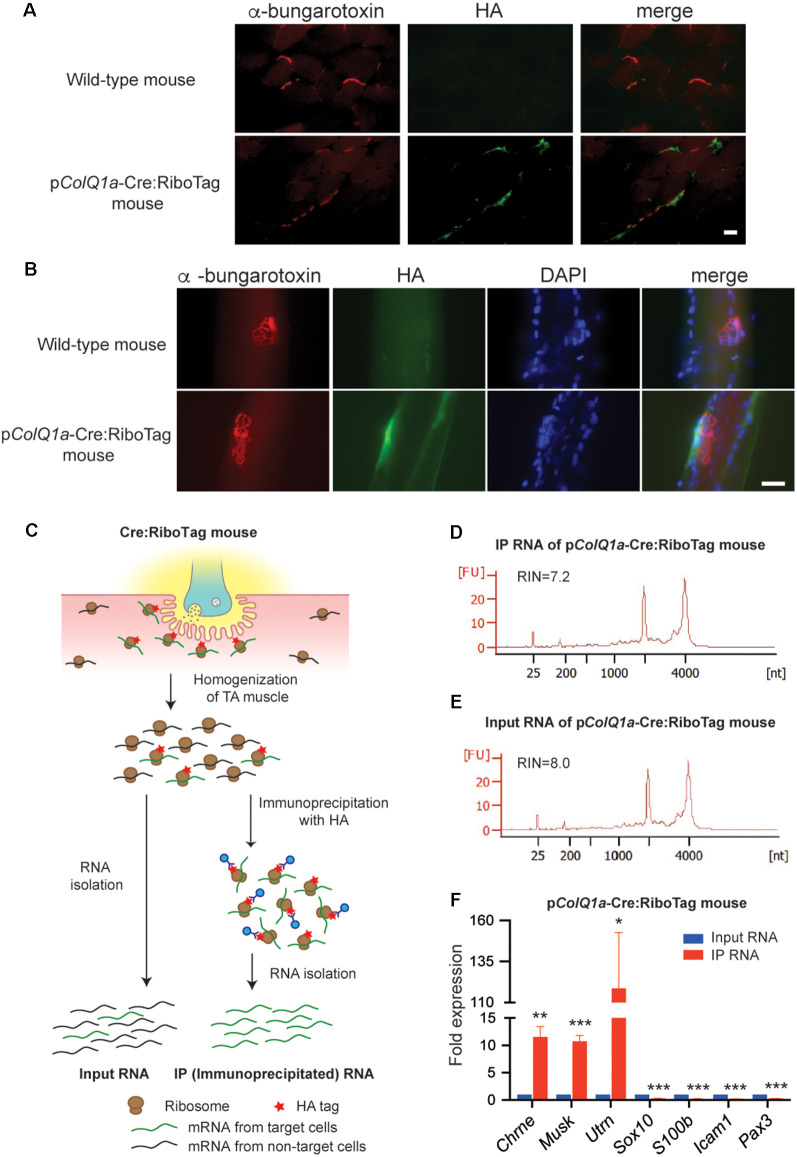
p*ColQ1a*-Cre:Ribotag mouse to disclose a gene expression profile at the motor endplate TA muscle. **(A,B)** Immunofluorescence staining of TA muscle of wild-type and p*ColQ1a*-Cre:RiboTag mice. Muscle sections **(A)** and teased muscle fibers **(B)** are stained with anti-HA antibody (green) and Alexa594-conjugated α-bungarotoxin 594 (red). Nuclei are stained with DAPI. Scale bar = 15 μm. **(C)** Schematic of the RiboTag technique for gene expression profiling at the motor endplate of TA muscle. **(D,E)** Agilent Technologies 2100 Bioanalyzer PicoChip outputs showing the quality of immunoprecipitated (IP) RNA **(D)** and input RNA **(E)**. **(F)** qRT-PCR of NMJ-specific *Chrne*, *Musk*, and *Utrn* genes, Schwann cell-specific *Sox10* and *S100b*, vascular endothelial cell-specific *Icam1*, and muscle satellite cell-specific *Pax3*. Gene expression level in IP RNA was normalized to that of *Gapdh*, and also to the ratio in input RNA. TA muscles of 10, 11, 12, and 16 p*ColQ1a*-Cre:Ribotag mice were pooled into four independent samples, and were analyzed in quadruplicate (*n* = 4). Mean and SD is indicated. *p < 0.05, **p < 0.01 and ***p < 0.001 by paired Student’s* t*-test.

We next mated our p*ColQ1a*-Cre mouse with RiboTag mouse (Sanz et al., 2009). The generated p*ColQ1a*-Cre:Ribotag mouse should express HA-tagged ribosomal protein RPL22 specifically at the motor endplate of fast-twitch muscle. To verify that the HA-tagged ribosomes were expressed specifically at the NMJ in p*ColQ1a*-Cre:RiboTag mice, we immunostained the HA-tag in cross-sections of TA muscle (Figure 2A) and teased TA muscle fibers (Figure 2B). Staining for the HA-tag was mostly overlapped with α-bungarotoxin-stained AChR in cross-sections of TA muscle (Figure 2A) and teased TA muscle fibers (Figure 2B).

### Enrichment of NMJ-Specific Genes and Paucity of Genes in Other Cell Types in TA Muscle of p*ColQ1a*-Cre:Ribotag Mouse

To identify gene expression profiles at the motor endplate of TA muscle, we immunoprecipitated mRNA under translation using an anti-HA antibody, which was compared with input RNA (Figure 2C). We obtained ~30 ng of immunoprecipitated (IP) RNA and ~200 ng of input RNA by pooling TA muscles of 11 p*ColQ1a*-Cre:RiboTag mice. As a control, we confirmed that only 0.67 ng RNA was immunoprecipitated by anti-HA antibody by pooling TA muscles of eight wild type mice. We confirmed that the RNA integrity numbers (RIN), representing the quality of RNA, of the IP and input RNA were more than 7.0 (Figures 2D,E). We first verified that expressions of *Chrne*, *MuSK*, and *Utrn* that should be concentrated in the subsynaptic nuclei were enriched in IP RNA (Figure 2F). In contrast, markers for Schwann cells (*Sox10* and *S100b*), vascular endothelial cells (*Icam1*), and muscle satellite cells (*Pax3*) were markedly attenuated in IP RNA (Figure 2F). Therefore, IP RNA of TA muscles of p*ColQ1a*-Cre:RiboTag mice were highly enriched in motor endplate-specific genes with scarce contamination of genes from adjacent cells at the NMJ.

### Differential Gene Expression (DGE) Analysis Reveals Genes That Are Up- or Down-Regulated at the Motor Endplate of TA Muscle

**Figure 3:**
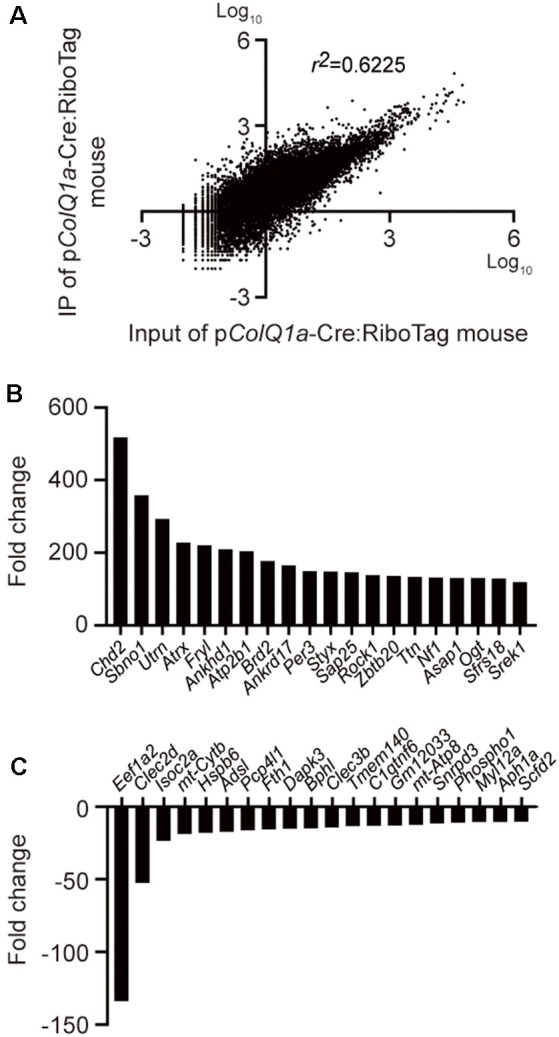
Differentially expressed genes between IP RNA and input RNA of the TA muscle. **(A)** Scatter plot of gene expressions in transcripts per million (TPM) between IP RNA and input RNA. The coefficient of determination (*r^2^*) is indicated. Representative upregulated **(B)** and downregulated **(C)** genes that were ranked top 20 each.

To extensively characterize a gene expression profile under translation at the motor endplate of TA muscle, we performed RNA-seq analysis of IP RNA and input RNA. A scatter plot of transcripts per million (TPM) of 22,383 protein-coding genes in IP RNA and input RNA showed that the coefficient of determination (*r^2^*) was 0.6225 (Figure 3A). As have been previously applied in other studies (Srivastava et al., 2017; Wang et al., 2019), we arbitrarily filtered genes with TPM >0.5 and fold change (IP/input or input/IP) >10. Differential gene expression (DGE) analysis identified 1177 genes: 1153 genes were upregulated and 24 genes were downregulated in the subsynaptic nuclei of TA muscle. The top 20 upregulated genes and the top 20 downregulated genes are graphically shown in Figures 3B,C, respectively.

### Gene Set Enrichment Analysis (GSEA) Reveals Key Gene Sets That Are Enriched at the Motor Endplate of TA Muscle

**Figure 4:**
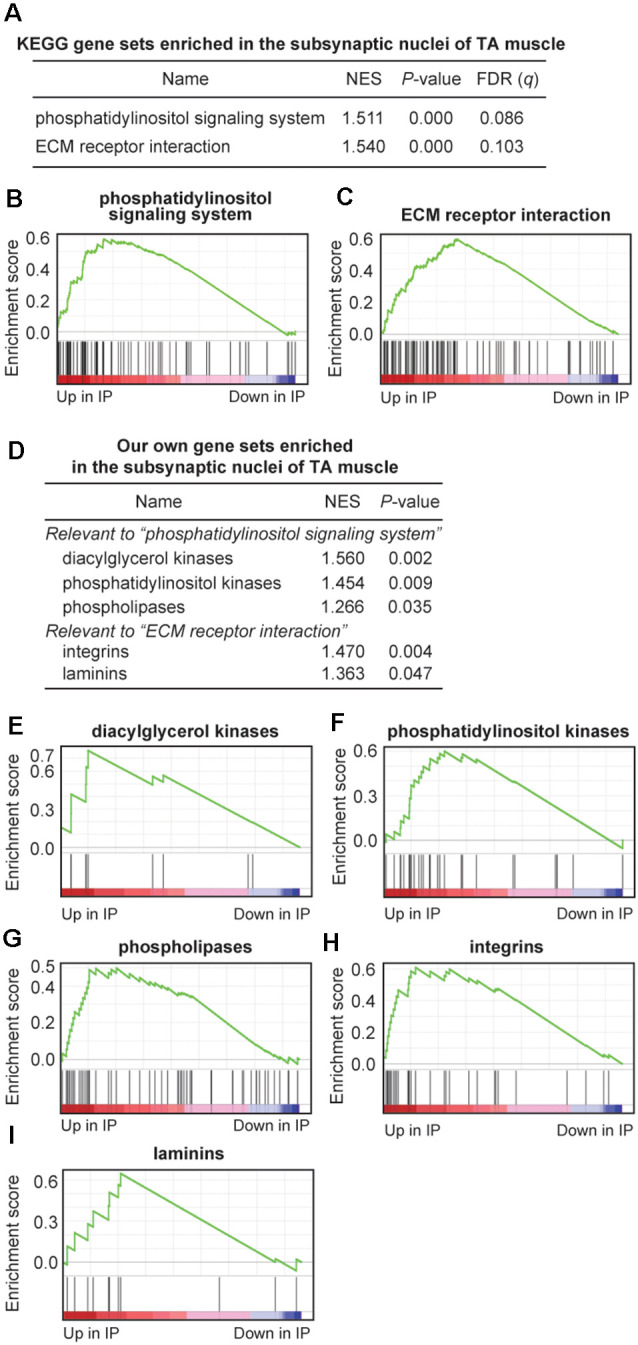
Gene sets enriched at the motor endplate of TA muscle by Gene Set Enrichment Analysis (GSEA) analysis. **(A–C)** Two KEGG gene sets were enriched at the motor endplate of TA muscle, which exceeded the thresholds of nominal *p*-value < 0.05 and false-discovery rate (FDR) *q*-value < 0.25. NES, normalized enrichment score of GSEA. **(D–I)** Enrichment at the motor endplate of TA muscle of our gene sets inspired by the two KEGG gene sets **(A)**. Our own gene sets are not subsets of the two KEGG gene sets but include additional relevant genes. See Supplementary Table S5 for gene names and TPM values included in each gene set.

To further characterize gene sets that were specifically enriched at the motor endplate of TA muscle, we performed GSEA using 186 gene sets retrieved from the Kyoto Encyclopedia of Genes and Genomes (KEGG) database. We found that two gene sets of “phosphatidylinositol signaling system” and “ECM receptor interaction” were enriched at the motor endplate (nominal *p* < 0.05, FDR *q* < 0.25; Figures 4A–C and Supplementary Table S5). We also made a set of genes that are expressed at the motor endplate and are associated with myasthenia gravis and postsynaptic congenital myasthenic syndromes such as *Chrne*, *Musk*, and *Dok7*. GSEA analysis showed that these genes were enriched at the motor endplate of TA muscle but without statistical significance, which was likely due to the limited number of genes (*p* = 0.070, Supplementary Figure S1 and Supplementary Table S5). The gene set “phosphatidylinositol signaling system” included genes encoding diacylglycerol kinases (DGK*), phosphatidylinositol kinases (PIK*), and phospholipases (PL*). Similarly, the gene set “ECM receptor interaction” included genes encoding integrins (ITG*) and laminins (LAM*). We made our own gene sets for the five gene families and performed GSEA analysis, and found that these genes were indeed enriched at the motor endplate of TA muscle (Figures 4D–I and Supplementary Table S5).

## Discussion

We first confirmed that p*ColQ1a* drives the expression of NLS-tagged Cre recombinase specifically at the motor endplate of TA muscle (Figure 1), and made a transgenic p*ColQ1a*-Cre mouse line. We then mated RiboTag mouse with the p*ColQ1a*-Cre mouse to make p*ColQ1a*-Cre:RiboTag mouse, which expressed an HA-tagged RPL22 specifically at the motor endplate of TA muscle (Figure 2) and enabled NMJ-specific gene expression profiling. mRNA under translation at the motor endplate was co-immunoprecipitated by an anti-HA antibody (Figure 2C). We confirmed that motor endplate-specific *Chrne* and *Musk* were enriched in IP RNA, whereas Schwann cell-specific *Sox10* and *S100b*, vascular endothelial cell-specific *Icam1*, and muscle satellite cell-specific *Pax3* were much reduced in IP RNA (Figure 2F). We then analyzed IP RNA by RNA-seq. We compared IP RNA with input RNA obtained from whole TA muscle to characterize motor endplate-specific gene expression profile of TA muscle (Figures 3, 4).

The mechanisms of NMJ-specific transcriptions of *ColQ* has been previously reported. Laser capture microdissection of the mouse NMJ revealed NMJ-specific expression of *ColQ* in the extraocular muscles (Ketterer et al., 2010) and the TA muscle (Nazarian et al., 2005; Ketterer et al., 2010). The NMJ-specific expression of *ColQ* is mediated by transcriptional upregulation at the NMJ and transcriptional downregulation in the extra-junctional regions. The E-box (CANNTG) in p*ColQ1a* binds to the myogenic differentiation factors (MDFs) of the MyoD family and drives muscle-specific gene expression (Lee et al., 2004). Contrarily, muscle action potentials suppress the E-box at the extra-junctional regions to drive NMJ-specific expression of an E-box-bearing gene (Ting et al., 2005). Transcription of most muscle-specific genes, however, are not suppressed by electrical activity, and other unidentified *cis*-elements are likely to be operational to achieve electrical transcriptional suppression (Ohno et al., 1999). Transcriptional upregulation of p*ColQ1a* at the NMJ is mediated by the N-box (CCGGAA; Lee et al., 2004). The N-box (CGGAA) is also the neuregulin-response element in mouse *Chrnd* encoding the δ subunit of acetylcholine receptor (AChR; Fromm and Burden, 1998) and in rat *Chrne* encoding the AChR ε subunit (Sapru et al., 1998). Similar involvement of the neuregulin-responsive N-box in the NMJ-specific transcription of *ColQ* in mouse have also been reported (Lee et al., 2004; Ting et al., 2005). Also, specific activation of p*ColQ1* and p*ColQ1a* in slow- and fast-twitch muscles, respectively, is mediated by a slow upstream regulatory element (SURE) in p*ColQ1* and a fast intronic regulatory element (FIRE) in p*ColQ1a* (Lee et al., 2004; Ting et al., 2005).

Cell-type-specific expression analysis has gained popularity in the past decades and is of particular importance to understand the biology of certain cell types or structures. The NMJ is one such complicated example consisting of presynaptic, synaptic, and postsynaptic components within a complex tissue environment (Slater, 2017; Huang et al., 2019). As stated above, NMJ-specific gene expression profiles have been analyzed by laser capture microdissection of extraocular muscles (Ketterer et al., 2010) and TA muscle (Nazarian et al., 2005; Ketterer et al., 2010), which, however, were inevitably contaminated with vascular endothelial cells, lymphocytes, and terminal Schwann cells. p*ColQ1a*-Cre:RiboTag mouse enabled extensive profiling of gene expressions under translation at the motor endplate of TA muscle with minimal contamination of adjacent cells. Gene expression profiling with RiboTag mouse inherently relies on the tissue- or cell-type-specific expression of HA-tagged RPL22 (Sanz et al., 2009). Although p*ColQ1a* was likely to be specifically active at the motor endplate of TA muscle, the transgenic p*ColQ1a*-Cre mouse carried a 3519-bp fragment of the *ColQ1a* promoter, which should have allowed a small amount of leaky expression of Cre recombinase at extra-junctional regions. A combination of laser capture microdissection and RiboTag may elucidate more precise gene expression profiles of the NMJ.

GSEA analysis revealed that two gene sets of “phosphatidylinositol signaling system” and “ECM receptor interaction” in the KEGG database were enriched at the subsynaptic nuclei of TA muscle (Figures 4A–C). Inspection of the two upregulated gene sets prompted us to make our own gene sets that covered more relevant genes than the KEGG database. Being prompted by the gene set of “phosphatidylinositol signaling system,” we found that genes encoding diacylglycerol kinases, phosphatidylinositol kinases, and phospholipases were enriched at the motor endplate of TA muscle (Figures 4D–G). Diacylglycerol kinases (DGK) are enzymes that phosphorylate diacylglycerol to yield phosphatidic acid. In the adult mouse, DGK-ζ encoded by *DGKZ* binds to syntrophin on the sarcolemma and is concentrated at the NMJ in a dystrophin-independent manner (Abramovici et al., 2003). DGK-ζ is likely to control lipid-signaling pathways to regulate actin organization (Abramovici et al., 2003). In *C. elegans*, presynaptic diacylglycerol facilitates the release of acetylcholine at NMJ, and DGK possibly regulates the abundance of presynaptic diacylglycerol (Lackner et al., 1999). We showed that DGK-η (*DGKH*) and DGK-θ (*DGKQ*) were 140-fold and 27-fold enriched at the motor endplate, respectively, and were more enriched than DGK-ζ (*DGKZ*; Supplementary Table S5). These and other DGKs may have undisclosed roles at the motor endplate. Phospholipases are enzymes that hydrolyze phospholipids into inositol phosphates and diacylglycerol, which are implicated as second messengers for the subsequent cellular events (Kadamur and Ross, 2013). Phospholipase C (PLC), one of the major classes of phospholipases, evokes endplate responses to acetylcholine in rodent skeletal muscle (Harborne et al., 1987). Two other classes of phospholipases are likely to have presynaptic roles. Phospholipase D blocks acetylcholine release by reducing the number of active presynaptic-releasing sites (Humeau et al., 2001). Phospholipase A_2_ regulates acetylcholine release with an inhibitory or excitatory effect in several distinct phases by controlling the Ca^2+^ or K^+^ channels (Fathi et al., 2001). We showed that phospholipases D and A_2_ are also enriched at the motor endplate (Supplementary Table S5), and these phospholipases may exert their effects by signaling through the motor endplate to the nerve terminal or by exerting undisclosed roles at the motor endplate. Similarly, by being prompted by the gene set of “ECM receptor interaction,” we found that genes encoding integrins and laminins were enriched at the motor endplate of TA muscle (Figures 4D,H,I). Integrins are cell surface receptors that facilitate adhesion of ECM molecules. The roles of integrins at the NMJ have been analyzed for integrins α3 and α7β1. Integrin α3 regulates multiple aspects of the development, function, and maintenance of the NMJ (Ross et al., 2017). Integrin α3 is required for fully efficient neurotransmission at the NMJ and also for the connection of motor neurons to the muscle fibers (Ross et al., 2017). Integrin α7β1 is concentrated at the postsynaptic NMJ in human and mouse, and their abundance is slightly increased in *mdx* mice in response to dystrophin deficiency (Hodges et al., 1997). We showed that yet uncharacterized integrins α2b and α10 were 163-fold and 68-fold enriched at the motor endplate (Supplementary Table S5). These and other integrins may have essential effects on the NMJ. Some laminins are also enriched at the NMJ (Rogers and Nishimune, 2017). Laminins α5 and β2 are enriched at the synaptic basal lamina, and laminin α4 is surrounding the nerve terminal, whereas laminins α2, β1, and γ1 are ubiquitously expressed on muscle fibers (Nishimune et al., 2008; Singhal and Martin, 2011). We found that laminins α3 and γ3 were 25-fold and 6-fold enriched at the motor endplate, respectively (Supplementary Table S5), and these laminins may have yet undisclosed roles at the NMJ.

To summarize, we have determined a gene expression profile at the motor endplate of TA muscle, which was expected to be minimally contaminated with adjacent cells at the NMJ. We expect that our gene expression profiling will contribute to the understanding of the molecular mechanisms of development, maintenance, and pathology of the NMJ.

## Data Availability Statement

FASTQ files are available at the DNA Data Bank of Japan (DDBJ) under the accession number DRA010277 (https://www.ncbi.nlm.nih.gov/sra/?term=DRA010277).

## Ethics Statement

The animal study was reviewed and approved by Animal Care and Use Committee of the Nagoya University Graduate School of Medicine.

## Author Contributions

KO conceived the project. KH, MI, and KO designed experiments. KH and JL performed most of the experiments with the help of MI, BO, and AM. RNA-seq analysis was performed by J-IT and TO. KH, MI, and KO wrote the article.

## Conflict of Interest

The authors declare that the research was conducted in the absence of any commercial or financial relationships that could be construed as a potential conflict of interest.

## Footnotes

^1^http://software.broadinstitute.org/gsea/index.jsp

## Supplementary Material

FIGURE S1 GSEA analysis of a set of genes that are associated with myasthenia gravis and postsynaptic congenital myasthenic syndromes. See Supplementary Table S5 for gene names and TPM values included in the gene set.

TABLE S1 Primer sequences for plasmid construction and genotyping.

TABLE S2 Primer sequences for qRT-PCR.

TABLE S3 The Numbers of Cre-positive and Cre-negative NMJs in teased TA muscle fibers electroporated with pCMV-Cre or p*ColQ1a*-Cre.

TABLE S4 Distribution of Cre-positive signals in teased muscle fibers of the TA and soleus muscles of the p*ColQ1a*-Cre mouse.

TABLE S5 Detailed values of RNA-seq analysis. TPM of 22,382 genes in input RNA and IP RNA. Results of GSEA analysis of two KEGG gene sets and five our own gene sets that were enriched at the motor endplate of TA muscle.
