# Supplementary figures and images for: Gene Expression Profile at the Motor Endplate of the Neuromuscular Junction of Fast-Twitch Muscle
Source: Front Mol Neurosci. 2020 Sep 8;13:154. doi: 10.3389/fnmol.2020.00154 (PMC7549434; doi:10.3389/fnmol.2020.00154)

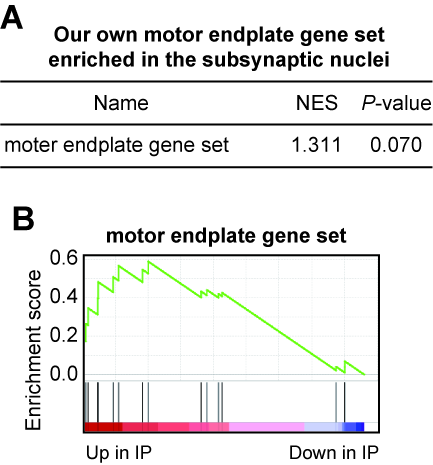

Supplement: FIGURE S1 — GSEA analysis of a set of genes that are associated with myasthenia gravis and postsynaptic congenital myasthenic syndromes. See Supplementary Table S5 for gene names and TPM values included in the gene set. [file Image_1.TIF]
