# Supplementary material for: Gene Expression Profile at the Motor Endplate of the Neuromuscular Junction of Fast-Twitch Muscle
Source: Front Mol Neurosci. 2020 Sep 8;13:154. doi: 10.3389/fnmol.2020.00154 (PMC7549434; doi:10.3389/fnmol.2020.00154)
Supplement: TABLE S1 — Primer sequences for plasmid construction and genotyping. [file Data_Sheet_1.docx]

**Supplementary Table S1. Primer sequences for plasmid construction and genotyping**

| **Name of Primers** | **Primer Sequence 5’-3’** |
| --- | --- |
| Cre-F-XhoI for cloning | ATACTCGAGCACCATGTCCAATTTACTGAC |
| Cre-R-XbaI for cloning | CAGTCTAGACTAATCGCCATCTTCCAGCAG |
| pCMV-F-BamHI for cloning | CCCGGTACCACGCGTACTAGTTATTAATAG |
| pCMV-R-XhoI for cloning | AAACTCGAGGGAGGCTGGATCGGTCCCGGT |
| pColQ1a-F-BamHI for cloning | CTAGGTACCTCACTTTCTTGGCAGGTGTGT |
| pColQ1a-R-XhoI for cloning | GCACTCGAGCTGTTCAGAGAAAACTGCTG |
| P1 for genotyping | CAGATCGTCATCCTAACTCCATGCTAAG |
| P2 for genotyping | GTACGTGAGATATCTTTAACCCTGATCCTG |

Restriction sites are underlined.

**Supplementary Table S2. Primer sequences for qRT-PCR**

| **Gene** | **Forward primer (5'-3')** | **Reverse primer (5'-3')** |
| --- | --- | --- |
| *Gapdh* | ACCCCTTCATTGACCTCAAC | TCCCGTTGATGACAAGCTTC |
| *Chrne* | TCACCCTAACCAACCTCATCTC | ATCGTCCTTGCTGTAGTTGAGC |
| *Musk* | AAGGCTACTGTGCCCAGTACAG | CAGCTCATTCCACGCAGTG |
| *Utrn* | GCCATCATCTTGGTGAATGCTCG | GGATGAAGGGTCCTGACCAATC |
| *Sox10* | GACGATGACAAGTTCCCCGT | GGCTAGCTTTCTGCGTGCC |
| *S100b* | AGAGGGTGACAAGCACAAGCTG | GAACTCCTGGAAGTCACACTCC |
| *Icam1* | AAACCAGACCCTGGAACTGCAC | GCCTGGCATTTCAGAGTCTGCT |
| *Pax3* | GCGTCTCTAAGATCCTGTGCAG | GATTTCCCAGCTAAACATGCCCG |

**Supplementary Table S3. The numbers of Cre-positive and Cre-negative NMJs in teased TA muscle fibers electroporated with pCMV-Cre or p*ColQ1a*-Cre**

|  | pCMV | p*ColQ1a* |
| --- | --- | --- |
| Cre-positive NMJs | 17 | 127 |
| Cre-negative NMJs | 119 | 20 |
| Ratio of Cre-positive NMJs | 12.5% | 86.4% |

The numbers of Cre-positive NMJs and Cre-negative NMJs were counted in teased TA muscle fibers in 4 pCMV-Cre mice (*n* = 136 fibers) and 5 pColQ1a-Cre mice (*n* = 147 fibers). *P* < 0.0001 by Fisher’s exact test.

**Supplementary Table S4. Distribution of Cre-positive signals in teased muscle fibers of the TA and soleus muscles of p*ColQ1a*-Cre mouse.**

|  | NMJ | NMJ & non-NMJ | non-NMJ | unstained |
| --- | --- | --- | --- | --- |
| TA muscle | 68 (63.6%) | 24 (22.4%) | 6 (5.6%) | 9 (8.4%) |
| Soleus muscle | 15 (14.9%) | 8 (8.0%) | 25 (24.8%) | 53 (52.5%) |

The numbers of Cre-positive signals along teased muscle fibers isolated from TA (*n* = 107 fibers) and soleus (*n* = 101 fibers) muscles. The ratio of teased muscle fibers is shown in parentheses. *P* < 0.001 by Fisher’s exact test.
